# Accrued reductions in heart rate following transcutaneous vagal nerve stimulation in adults with posttraumatic stress disorder

**DOI:** 10.3389/fnins.2025.1456662

**Published:** 2025-03-28

**Authors:** Srirakshaa Sundararaj, Asim H. Gazi, Viola Vaccarino, Amit J. Shah, Omer T. Inan, J. Douglas Bremner

**Affiliations:** ^1^Edward Via College of Osteopathic Medicine, Spartanburg, SC, United States; ^2^College of Sciences, Georgia Institute of Technology, Atlanta, GA, United States; ^3^School of Engineering and Applied Sciences, Harvard University, Cambridge, MA, United States; ^4^School of Electrical and Computer Engineering, Georgia Institute of Technology, Atlanta, GA, United States; ^5^Department of Epidemiology, Rollins School of Public Health, Emory University, Atlanta, GA, United States; ^6^Department of Internal Medicine, Emory University School of Medicine, Atlanta, GA, United States; ^7^Atlanta VA Medical Center, Decatur, GA, United States; ^8^Coulter Department of Biomedical Engineering, Georgia Institute of Technology, Atlanta, GA, United States; ^9^Department of Psychiatry and Behavioral Sciences, Emory University School of Medicine, Atlanta, GA, United States; ^10^Department of Radiology and Imaging Sciences, Emory University School of Medicine, Atlanta, GA, United States

**Keywords:** Post-Traumatic stress disorder (PTSD), transcutaneous cervical vagus nerve stimulation, heart rate, neuromodulation, autonomic nervous system

## Abstract

**Background:**

Post-Traumatic Stress Disorder (PTSD) is a condition marked by chronic autonomic dysregulation, including heightened arousal and increased heart rate, contributing to a higher risk of cardiovascular disease (CVD). tcVNS has been shown in prior work to decrease arousal and reduce heart rate in participants with PTSD during stimulation and 2–3 min afterward. No studies have investigated effects of tcVNS on objective physiological markers such as heart rate over hour-long timescales.

**Objective:**

The purpose of this study was to investigate the effects of active versus sham tcVNS on heart rate responses to stress in traumatized individuals with and without PTSD undergoing a 3 h traumatic stress reminder protocol. Understanding the effects of tcVNS on heart rate over extended periods lasting several hours is crucial to better assess its potential cardiovascular benefits and inform treatment strategies for this population.

**Methods:**

A total of 41 participants, including 22 with PTSD (sex: 16 female, six male; age: 41.5 ± 12.89 years) and 19 without (sex: nine female, 10 male; age: 32.79 ± 7.10 years), participated in a mechanistic clinical trial that elicited trauma-incited stress responses by replaying recorded traumatic scripts followed by active or sham tcVNS (double-blind, randomized). Continuous electrocardiogram data were collected and used to measure heart rate and temporal alignment and resampling of the beat-by-beat heart rate time series were performed to distinctively enable timepoint by timepoint averaging for the entire 3 h research visit. We then aggregated the responses across participants of the same group (active/sham × PTSD/non-PTSD) and investigated the effects of tcVNS on heart rate over the multi-hour protocol.

**Results:**

Analysis revealed accrued reductions in heart rate among participants with PTSD who received active tcVNS compared to those who received sham stimulation (*P* < 0.05). These effects were not observed in non-PTSD participants. Notably, heart rate reduced approximately 5% below baseline levels for participants with PTSD who received active tcVNS by the end of the ∼3 h-long protocol, indicating accrued effects of tcVNS on cardiac autonomic function.

**Conclusion:**

tcVNS induces accrued reductions in heart rate for participants with PTSD, potentially suggesting a temporary reduction of chronic cardiac arousal associated with PTSD.

## Introduction

Post-Traumatic Stress Disorder (PTSD) is a debilitating condition typically triggered by traumatic experiences such as instances of abuse, military combat, natural disasters, and accidents (Bryant, 2019; Lancaster et al., 2016; Morganstein et al., 2021; Watkins et al., 2018). Individuals with PTSD often endure chronic and exaggerated autonomic responses to stress, characterized by heightened arousal states (Gurel et al., 2020b; Lamb et al., 2017). These responses encompass a spectrum of physiological changes, including increased heart rate, thereby distinguishing PTSD sufferers from those without the disorder (Dennis et al., 2014; Haag et al., 2019; Sadeghi et al., 2022). Previous research has identified correlations between lower HRV and cardiovascular dysregulation, highlighting the connection between untreated PTSD and the onset of heart disease and elevated mortality rates (Ehret, 2019; Fu, 2022). Furthermore, evidence suggests that individuals with PTSD are at a higher risk for cardiovascular disease (CVD), and the physiological stress responses associated with PTSD may contribute to this elevated risk (O’Donnell et al., 2021; Sumner et al., 2023; Turcu et al., 2023). This interplay between autonomic dysregulation and cardiovascular health underscores the multifaceted nature of PTSD. Addressing PTSD’s physiological manifestations may require interventions targeting its physiological manifestations (Ehret, 2019).

Given the strong correlation between autonomic dysfunction and CVD in PTSD participants at the group level, it is crucial to explore therapeutic interventions that can mitigate these physiological abnormalities. The appeal of transcutaneous cervical vagus nerve stimulation (tcVNS) lies in its ability to modulate central and autonomic nervous system pathways implicated in stress response and emotional regulation, offering a novel avenue for symptom management. tcVNS has demonstrated efficacy in mitigating stress-induced physiological changes across a spectrum of markers (Bremner et al., 2021; Bremner et al., 2020a; Bremner et al., 2020b; Choudhary et al., 2023; Doerr et al., 2023; Gazi et al., 2021a; Gazi et al., 2021b; Gazi et al., 2022b; Gurel et al., 2020a; Gurel et al., 2020b; Moazzami et al., 2023; Wittbrodt et al., 2020, 2021). In particular, research has shown that tcVNS can effectively modulate peripheral physiological markers of the autonomic nervous system such as heart rate, heart rate variability, respiration rate, respiratory variability, vascular tone, and others. The primary analyses of Gurel et al. (2020a,b) in 2020 demonstrated that tcVNS reduced peripheral physiological markers of sympathetic arousal and parasympathetic withdrawal for individuals with history of prior trauma in comparison to sham stimulation. The two trials were conducted in a double-blind, randomized, sham-controlled manner, where tcVNS-induced changes were observed in participants without PTSD (Gurel et al., 2020a), as well as for individuals with PTSD (Gurel et al., 2020b). The changes observed were quantified during tcVNS and a few minutes after tcVNS administration by averaging over minutes at a time and over multiple repetitions of tcVNS. The time course of peripheral physiological changes during and after tcVNS was not investigated in the primary analyses of Gurel et al. (2020a,b) nor were the effects of tcVNS over the course of the entire protocol that lasted hours.

**FIGURE 1:**
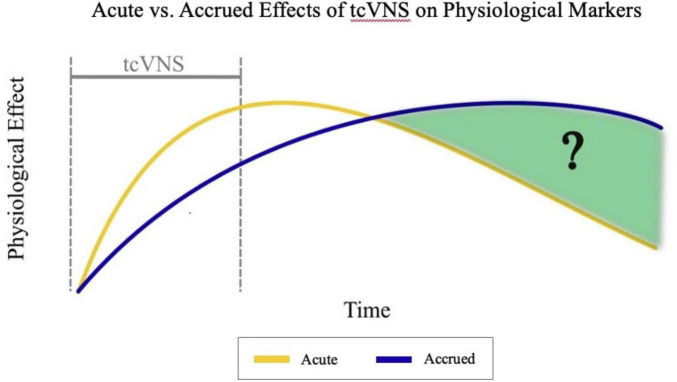
Hypothesized comparison between acute and accrued effects of transcutaneous cervical vagus nerve stimulation (tcVNS) over time. tcVNS causes an acute impact on physiological markers initially. The magnitude of this effect decreases over time at a relatively faster rate than the accrued effects asserted by this analysis. The accrued effects increase initially, and these effects are endured longer than the acute effects.

The time course of peripheral physiological changes in response to tcVNS and whether changes accrue over time following stimulation remains understudied. Prior secondary analyses of the same data have investigated dynamic changes in physiological markers such as heart rate during tcVNS, analyzing responses a few minutes before, during, and after the stimulation period (Gazi et al., 2020, 2023). However, the effects of tcVNS on heart rate over longer timescales and across multiple stimulation sessions have not been investigated. As shown in Figure 1, our current work addresses this previously unanswered question and investigates the time course of heart rate effects following repeated tcVNS over the course of hours by analyzing the data collected in prior work (Gurel et al., 2020a,b). We hypothesized that individuals with history of psychological trauma who receive tcVNS will exhibit a prolonged reduction in heart rate compared to those receiving sham stimulation.

## Materials and methods

### Study protocol

Our analysis focuses on the first morning of a 3 days study (ClinicalTrials.gov NCT02992899) which received approval from the Institutional Review Boards (IRB) at Georgia Institute of Technology (GT, #H17126), Emory University School of Medicine (Emory, #IRB00091171), SPAWAR Systems Center Pacific, and the Department of Navy Human Research Protection Program. This subset of the data was chosen to mitigate potential confounding effects from meal intake and circadian rhythm variability. A total of 50 participants were recruited for the study. Nine participants were excluded from the analysis due to missing data resulting from early termination of data collection. Individuals with cardiovascular conditions that could influence autonomic function, such as carotid atherosclerosis, were excluded to prevent confounding effects on heart rate regulation (Gurel et al., 2020a; Turcu et al., 2023). The remaining 41 subjects had sufficient data for analysis, but outliers with missing data due to early termination were not included to ensure the integrity of the statistical analysis. Note that our analysis, unlike prior analyses of this data, requires the majority of data for a participant to be present to analyze changes over the course of the protocol. The 41 participants comprised of 22 individuals diagnosed with PTSD (11 active tcVNS, 11 sham tcVNS) and 19 non-PTSD individuals (11 active tcVNS, 8 sham tcVNS). Non-PTSD individuals each had a history of psychological trauma but participated in our study without a PTSD diagnosis. Participants experienced a range of prior traumas, including combat, motor vehicle accidents, abuse, and assaults. For additional recruitment and study cohort details, please refer to Gurel et al. (2020a) for non-PTSD participants and (Gurel et al., 2020b) for participants with PTSD.

The experimental environment was maintained at approximately 20°C between the hours of 9:00 and 13:00. Physiological signals were collected for approximately two to three of those hours. Participants were instructed to abstain from consuming caffeine throughout the entirety of the protocol and to fast after 6:00. Individuals in the study were exposed to 10 stimuli including neutral audio scripts, trauma audio scripts, and active or sham tcVNS. The protocol began with two neutral scripts, then two traumatic scripts accompanied by tcVNS administration, then two stimulation-only conditions, followed by another two neutral scripts, and finally two traumatic scripts accompanied by tcVNS. Neutral scripts depicted pleasant scenes to evoke neutral or positive emotions, whereas trauma scripts comprised personalized narratives of past traumatic events. Each participant was exposed to four trauma scripts. These scripts were developed based on a combination of approaches: some scripts described distinct traumatic events, while others focused on multiple aspects or facets of a single traumatic event. Once the scripts were developed, a research associate narrated the scripts in a normal tone of voice, recording these narrations for playback during trauma recall (Bremner et al., 1999; Gazi et al., 2024; Orr et al., 2002). Participants listened to all scripts via headphones. Neutral and trauma scripts all lasted approximately 60 s.

The conditions were ordered to isolate and analyze the effects of tcVNS on both neutral and trauma recall responses, as well as to compare these effects in the absence of acute stressors. Beginning with neutral recordings allowed for the establishment of a baseline for physiological responses. Traumatic scripts were then introduced, immediately followed by stimulation to assess the immediate impact of tcVNS on stress-induced arousal. This was followed by stimulation without the trauma reminders to observe its direct effects. All participants were debriefed at the end of the protocol and, if needed, further evaluated by the study psychiatrist or referred for additional care to avoid psychic crisis due to trauma recall. For further details regarding the 10 conditions and their ordering, please refer to Supplementary Figure 1 and Gazi et al. (2022b).

### Double-blinding and randomization

Participants were randomly assigned to receive either active tcVNS targeting the left vagus nerve or sham stimulation. The active and sham devices utilized were gammaCore devices from electroCore (Basking Ridge, NJ, United States), which appeared and functioned identically. The only variable that differed between active and sham groups was the stimulation waveform. The active stimulation waveform consisted of five 5 kHz sinusoidal pulses repeating at a rate of 25 Hz. Sham stimulation entailed biphasic square pulses repeating at a rate of 0.2 Hz. Stimulation administrations lasted for exactly 120 s each following each trauma reminder during the four trauma and tcVNS conditions, as well as for the two standalone administrations. As outlined in the primary analysis, Gurel et al. (2020a), the 120 s stimulation duration was determined based on the recommended operation of the electroCore gammaCore device. This duration has also been established in prior work to elicit measurable autonomic effects (Gazi et al., 2020; Gazi et al., 2022a; Gazi et al., 2022b; Gurel et al., 2020a; Marano et al., 2024; Okdahl et al., 2024; Sigurdsson et al., 2021; Wittbrodt et al., 2021). For further details, please refer to the primary analysis, (Gurel et al., 2020a).

### Physiological sensing and feature extraction

Heart rate served as the primary physiological parameter of interest. Data were collected using the Biopac RSPEC-R system (Biopac Systems, Goleta, CA) and acquired via the Biopac MP150 system at a sampling rate of 2 kHz as described in Gurel et al. (2020a). Heart rate was extracted from the electrocardiogram (ECG) signal on a beat-by-beat basis, following the established methods described in Gazi et al. (2022b).

Unlike physiological markers that may require detrending due to external factors, heart rate can be directly interpreted in its absolute form. The relative changes analyzed in this study account for differences in absolute baseline heart rate across participants. Drifts in heart rate during longitudinal analysis are interpreted as physiological changes of interest rather than artifacts to be corrected. Detrending was not necessary for the heart rate data, as any observed drift in heart rate is integral to the findings of this study, rather than an aspect to be filtered out.

### Time series processing

**FIGURE 2:**
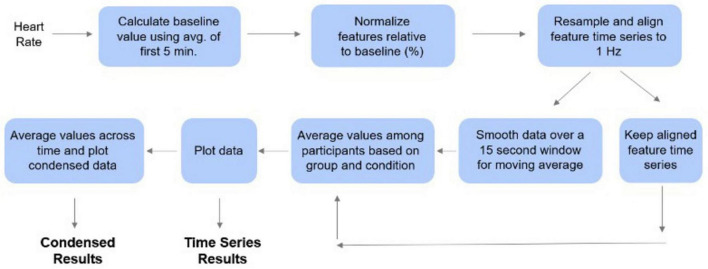
Processing pipeline for time series analysis. The first 5 min of data for each participant were used as a baseline to normalize the heart rate data. These values were then averaged among the participants. The data points were averaged for each condition to produce the condensed plots. The time series analysis was conducted by plotting each data point over the first four hours of data collection.

Baseline heart rate values were calculated by taking the average of the data from the first 5 min of the experimental session for each participant. Subsequently, each heart rate value was normalized relative to this baseline by subtracting and then dividing by the baseline (Lambert et al., 2024; Heckman et al., 2007; Mirmoosavi et al., 2024). Temporal alignment of the participants’ time series was achieved by linearly resampling the heart rate time series to a 1 Hz (1/s) frequency which allows for second-by second analysis. This was followed by timepoint-by-timepoint alignment via linear interpolation. These time series were retained, but for visualization purposes, we separately applied a 15 s moving average (centered on the datapoint) to the time series to visualize smoother trends see Figure 2.

### Analyses

#### Condensed plots (cure 3)

**FIGURE 3:**
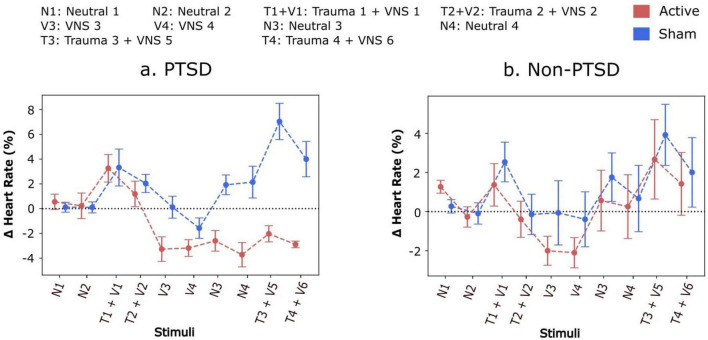
**(a,b)** Condensed comparison between relative change in heart rate for participants receiving either active or sham stimulation during each condition of protocol. Participants receiving active stimulation experienced a greater accrued decrease in their heart rate when compared to sham participants. On average, participants with Post-Traumatic Stress Disorder (PTSD) experienced a decrease in heart rate during the conditions with trauma recall and stimulation. Heart rate increases and returns above baseline for active and sham participants without PTSD and sham participants with PTSD during neutral scripts 3 and 4. However, heart rate remains below baseline during both neutral scripts 3 and 4. Furthermore, these effects remain even during trauma recall 3 and 4.

To first investigate the overall average values among participants within each experimental group and condition, a single average value was computed for each condition and then averaged across all participants within a group. These values were then plotted across time, along with their standard errors, for each condition to generate what we call “condensed plots”, as shown in Figure 3. The observation of decreased heart rate persisting beyond the stimulation period in PTSD participants prompted further investigation and encouraged a broader depiction of how heart rate is modulated within and between conditions. Specifically, the question of whether heart rate returned to baseline and then subsequently reduced during each stimulation or did heart rate reduce during stimulation and remain decreased afterward could not be answered with these condensed plots.

#### Time series plots

**FIGURE 4:**
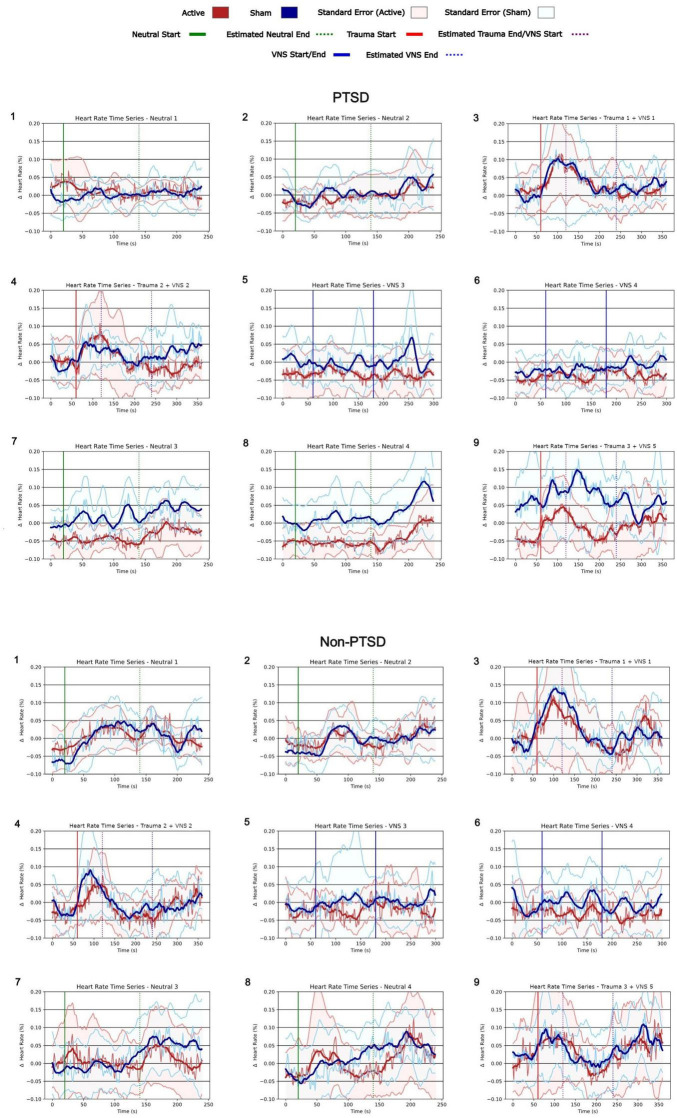
Time series analysis of relative changes in heart rate for Post-Traumatic Stress Disorder (PTSD) and non-PTSD participants during each condition of protocol. In the PTSD active group (red line), heart rate decreased during stimulation and remained below pre-stimulation levels thereafter, with no post-stimulation increases above baseline (indicated by 0.00 on the y-axis). During trauma recall in the third script, heart rate for the PTSD active group increased but returned to approximately baseline, contrasting with the PTSD sham group’s response (blue line). Participants without PTSD showed no significant differences between active (red line) and sham (blue line) groups.

Accordingly, time series plots, as shown in Figure 4 were generated to depict the heart rate dynamics over the entire protocol duration. This process involved aggregating and averaging the heart rate time series described in the previous section (see section “Time series processing”) across participants timepoint-by-timepoint or second-by-second. These averages, along with their standard errors, were plotted as a mean plus or minus standard error time series. A subplot was created for each experimental condition to depict the evolution of heart rate within and across the neutral, trauma, and tcVNS/sham conditions with respect to their average baseline or pre-stimulation heart rate. It should be noted that the number of subjects with sufficient data for Trauma Script 4 was insufficient to enable consistent time series alignment with the other scripts. This limitation impacted the comparability of the time series plots for Trauma Script 4 to the other experimental conditions leading Trauma 4 + VNS 6 to be excluded from these plots.

#### Statistical testing

For the PTSD and non-PTSD groups separately, a mixed analysis of variances (ANOVA) was conducted with two factors, a within-participants factor and a between-participants factor. The within-participants factor was the protocol condition (i.e., N1, N2, T1 + V1, etc.), and the between-participants factor was the active or sham grouping. Upon finding a statistically significant interaction effect for the PTSD group, two-sample *t*-tests or Mann-Whitney U tests (Shapiro-Wilk tests were used to assess normality) were conducted to further compare active and sham groups for each condition (corresponding to the condensed plots). These *post hoc* analyses were conducted to identify statistically significant differences between groups for each condition.

## Results

### Participant characteristics

Eleven of the participants with PTSD received sham stimulation (sex: six female, five male; age: 42.54 ± 13.59 years, BMI: 28.59). The other eleven participants with PTSD received active stimulation (sex: 10 female, one male; age: 40.36 ± 12.64 years, BMI: 28.69).

Eight of the participants without PTSD received sham stimulation (sex: five female, three male; age: 30.50 ± 3.51 years, BMI: 28.48). The other eleven participants without PTSD received active stimulation (sex: four female, seven male; age: 34.45 ± 8.65 years, BMI: 26.20). Refer to Supplementary Table 1 for further details.

### Condensed plots illustrate the effects of tcVNS on heart rate across all conditions

Condensed plots depicting the average effects of tcVNS during each condition are shown in Figure 3. Participants without PTSD and participants with PTSD are plotted separately, where active and sham subgroups are overlaid for ease of comparison. For participants with PTSD, the average heart rate relative to baseline fell to approximately -4% following repeated stimulation, compared to the sham group’s relative heart rate remaining at 0% or above. Specifically, for the final trauma condition, Trauma 4, the mean heart rate for the active tcVNS group was 0.014 (SD = 0.036), while the sham group exhibited a mean heart rate of 0.020 (SD = 0.061). No statistically significant differences between the active and sham groups for participants without PTSD were detected (main effect of active vs. sham: F = 0.14, *P* = 0.714, d = 0.01; main effect of protocol condition: F = 3.09, *P* = 0.002, d = 0.19; interaction with protocol condition: F = 0.17, *P* = 0.997, d = 0.01). Refer to Supplementary Table 2 for further details. On the other hand, the active vs. sham difference was statistically significant for the PTSD group, with a significant main effect of active vs. sham (F = 15.65, *P* = 0.001, d = 0.51), significant main effect of protocol condition (F = 4.34, *P* < 0.001, d = 0.22), and a significant interaction with protocol condition (F = 3.08, *P* = 0.002, d = 0.17). *Post hoc* analyses revealed statistically significant (*P* < 0.05) differences for the final four conditions of the protocol (Table 1), with no statistically significant differences for the protocol conditions beforehand. No significant differences were observed .

**TABLE 1:** Statistical analysis of mean relative change in heart rate for participants with PTSD.

Condition	Sham mean (SD)	Active mean (SD)	Test statistic	*P*-value
Neutral 1	0.001 (0.017)	0.006 (0.029)	t = 0.394	0.7
Neutral 2	0.001 (0.019)	0.002 (0.048)	U = 48	0.215^a^
Trauma 1 + VNS 1	0.033 (0.035)	0.033 (0.039)	U = 55	0.371^a^
Trauma 2 + VNS 2	0.02 (0.056)	0.012 (0.046)	t = 0.489	0.631
VNS 3	0.001 (0.065)	−0.033 (0.052)	t = 1.854	0.079
VNS 4	−0.016 (0.032)	−0.032 (0.048)	t = 1.113	0.279
**Neutral 3**	**0.019 (0.064)**	−**0.026 (0.031)**	**t = 2.871**	**0.01**
**Neutral 4**	**0.021 (0.062)**	−**0.037 (0.012)**	**t = 2.683**	**0.015**
**Trauma 3 + VNS 5**	**0.070 (0.039)**	−**0.020 (0.047)**	**t = 4.158**	**0.001**
**Trauma 4 + VNS 6**	**0.040 (0.036)**	−**0.029 (0.032)**	**t = 3.415**	**0.007**

^a^Mann Whitney U test was conducted instead of two-sample *t*-test due to non-normality. The mean relative change in heart rate from baseline [M (SD)] for the active and sham conditions is reported for each condition, along with the corresponding *p*-values and test statistics. Bolded values indicate comparisons that yielded statistically significant *p*-values (*p* < threshold).

### Time series plots elucidate tcVNS’s effects on heart rate within and across conditions

Time series plots shown in Figure 4 provide further insights into the accrued effects of tcVNS on heart rate dynamics for participants with PTSD. Heart rate is seen to decrease during stimulation and remain below pre-stimulation levels afterward for the active group. This is not apparent for the sham group. Notably, the decrease accrued to the extent that during the third trauma script, participants with PTSD in the active group experienced an acute increase in heart rate during trauma recall, but the increase returned heart rate to approximately 0% relative to baseline, rather than increase beyond that. This contrasts with what is observed for the sham group which remains at least 3% above baseline for the vast majority of the third trauma script. Participants without PTSD did not demonstrate these same differences between the active and sham groups. In the second trauma condition, Trauma 2, the mean heart rate for the active tcVNS group was −0.004 (SD = 0.077), compared to −0.001 (SD = 0.074) in the sham group supporting the lack of significant group differences in this population. As noted earlier, the time series data for Trauma Script 4 + VNS 6 could not be processed or time-aligned in the same manner as the other scripts due to limitations in data availability. Consequently, Trauma 4 + VNS 6 has been excluded from these plots.

## Discussion

### tcVNS induces a decrease in heart rate that accrues over time for participants with PTSD

Our results from a double-blind, randomized, controlled trial of tcVNS demonstrate that our hypothesis was partially correct. For participants with PTSD, tcVNS produces an accruing reduction in heart rate compared to sham stimulation. However, for traumatized individuals without PTSD, no accrued effects exist when comparing the active and sham groups.

The accrued decrease in heart rate observed in the active versus sham tcVNS conditions was evident both in the condensed plots and the time series plots for participants with PTSD. The condensed plots illustrate that comparing the active and sham groups, the active group’s heart rate values fell notably below the sham group’s following repeated stimulation. Statistical testing showed that this active vs. sham difference was significant for the final four conditions of the protocol. However, the condensed plots did not answer the question of whether heart rate simply decreased each time tcVNS was administered but returned to baseline afterward, or if tcVNS induced an accrued decrease in heart rate that persisted even after stimulation. The time series plots addressed this question. The time series plots illustrate how decreases in heart rate in the active group accrued over time in the active group compared to the sham group even after stimulation had finished. This accrued decrease remained until the final protocol condition, where during trauma recall, the participants with PTSD experienced heart rate increases, but their heart rate remained below their initial baseline (i.e., 0%) even during the trauma reminder. This suggests a protective effect induced by the cumulative effects of tcVNS, lowering an individual’s baseline autonomic arousal.

The unique application of time series processing in this analysis enabled us to answer questions related to time courses that previous studies were unable to address. By resampling and temporally aligning data across participants within a group, data could be averaged timepoint by timepoint. This enabled investigation of nuanced effects before and after stimulation that averaging would not allow to explore. Additionally, the use of time series processing provided added granularity to how heart rate decreased during tcVNS for participants with PTSD. Previous studies primarily examined the short-term effects of tcVNS on heart rate, showing transient reductions during stimulation for PTSD and non-PTSD participants (Gurel et al., 2020a,b). Specifically, Wittbrodt et al. detected reductions in heart rate during the second minute of stimulation and minutes afterward. However, the analysis of Gurel et al. (2020a) did not detect any difference in heart rate between the active and sham groups for the non-PTSD group during the second minute of stimulation or minutes afterward. Gazi et al. (2020) investigated the second minute of stimulation and minutes after stimulation for the non-PTSD group and found that tcVNS reduces heart rate for a brief period, moreso during the first minute, but the reduction in heart rate is transient and does not linger following stimulation (Gazi et al., 2020). These prior results are in agreement with our current findings. Our current findings show that tcVNS-induced reductions in heart rate linger on after stimulation and accrue following repeated stimulation for the PTSD group, with no evidence of similar, longer-term effects or accrual in non-PTSD participants. We thus took the initial results of Gazi et al. (2020) that were focused on effects during stimulation and extended them to the entirety of a data collection protocol to provide indication of whether the effects of tcVNS accrue following stimulation. This analysis represents an advancement in our understanding of the long-term effects of tcVNS on autonomic regulation.

Additionally, this analysis yields an observable difference between participants with PTSD and traumatized individuals without PTSD in terms of their heart rate responses to tcVNS. Individuals with PTSD experienced effects that accrue over time when compared to those without the disease. Previous work found that VNS may not be as effective for healthy individuals compared to those with PTSD (Sanchez-Perez et al., 2023). This distinction highlights the potential specificity of tcVNS effects on pathways unique to those with PTSD and suggests that its therapeutic benefits may be more pronounced in such populations with heightened autonomic arousal or dysregulation. Another potential explanation for these findings is that cardiovascular responses to trauma scripts are typically stronger in PTSD participants than in non-PTSD participants (Gurel et al., 2020b). This heightened physiological reactivity to trauma recall for individuals with participants with PTSD may have made it easier to detect changes induced by tcVNS, as participants with non-PTSD tend to exhibit less significant autonomic responses to trauma recall. Consequently, the accrued reductions in heart rate observed in PTSD participants likely reflect tcVNS’s ability to modulate these heightened responses over time, whereas the absence of significant effects in non-PTSD participants may be indicative of a more regulated baseline autonomic state for individuals without PTSD (Gurel et al., 2020b).

### Broader implications

The findings of this study may have implications for the management of cardiovascular symptoms associated with stress, anxiety, and hyperarousal in PTSD participants. TcVNS seems to exert an accruing modulatory influence on heart rate responses to trauma reminders, particularly in individuals diagnosed with PTSD. The observed reductions in heart rate post-stimulation, coupled with the attenuated response to trauma-induced arousal, highlight the potential therapeutic benefits of tcVNS in mitigating physiological hyperarousal associated with PTSD. Beyond its immediate autonomic effects, our findings suggest that repeated tcVNS administration may contribute to longer-term autonomic and cognitive regulation in PTSD. It has been demonstrated that tcVNS, when used consistently over a period of 3 months, enhances cognitive functions such as memory and attention in PTSD patientse (Choudhary et al., 2023). While our study primarily focused on acute effects on autonomic dysregulation over a single session, future work should explore whether prolonged tcVNS use leads to persistent autonomic recalibration and symptom reduction in PTSD, particularly in settings where participants can self-administer stimulation as part of daily therapy. Chronic hyperarousal and autonomic dysregulation are hallmark features of PTSD, characterized by an overactive sympathetic nervous system and underactive parasympathetic nervous system (Crawford et al., 2019; Fonkoue et al., 2020; Siciliano et al., 2022). The modulation provided by tcVNS may exhibit more pronounced effects in these participants to rebalance autonomic activity. Conversely, non-PTSD participants are less likely to experience such imbalances, resulting in more transient effects after stimulation.

The findings indicate that tcVNS does not influence the immediate increases in heart rate following trauma script administration. This implies that the modulatory effects of tcVNS on autonomic function may not protect against the acute heart rate spike triggered by trauma recall. Previous studies have demonstrated that tcVNS can reduce heart rate minutes after trauma recall (Gurel et al., 2020a,b). This study shows that in adults with PTSD, tcVNS may lead to an accruing reduction in heart rate, contributing to an overall lower heart rate response to trauma recall, even if the immediate response is not diminished. Furthermore, the latency of these effects suggests that tcVNS may engage slower biological pathways, such as hormonal or endocrine mechanisms, rather than eliciting immediate changes through rapid neurotransmitter actions. This delayed response may be explained by the interaction between tcVNS and the hypothalamic-pituitary-adrenal (HPA) axis (Fang et al., 2023; Li et al., 2020; Yan et al., 2024). While vagus nerve stimulation is well-documented to increase parasympathetic activity, its prolonged effects may also involve indirect regulation of the HPA axis through brainstem nuclei, including the nucleus tractus solitarius (NTS) (Olsen et al., 2023). The NTS serves as a primary relay center for afferent vagal input, influencing downstream autonomic and endocrine functions, including reductions in circulating cortisol levels and sympathetic tone. The extent to which tcVNS influences autonomic homeostasis through these pathways remains unclear, but the accruing reductions in heart rate observed in PTSD participants suggest that repeated stimulation may induce a shift toward sustained parasympathetic dominance. Engaging the slower pathways could help reset the body’s stress response system over time, offering a more enduring therapeutic benefit. This mechanism may underlie the accrued reduction in heart rate observed in PTSD patients, as hormonal modulation typically exerts its effects over hours rather than minutes, contrasting with the faster responses driven by neurotransmitter activity.

This reinforces the potential of tcVNS, with its accrued effects, to effectively modulate autonomic function and alleviate stress-related symptoms in PTSD participants. Moreover, the accrued effects of tcVNS on heart rate dynamics over time indicate potential that needs to be investigated more as to whether tcVNS can be used as a protective intervention for managing stress-related cardiovascular dysregulation. Longitudinal studies remain scarce but are necessary to investigate these implications (Bremner et al., 2021).

### Limitations and future work

Some limitations should be considered when interpreting the results of this study. Firstly, participants who withdrew early from the study were excluded from the analysis, potentially introducing selection bias. Additionally, some participants were excluded due to missing data, as our analysis required a majority of the protocol duration to be present for accurate time-course evaluation. This ensured that results reflected sustained autonomic changes rather than the influence of incomplete data points or outliers. Given that this study is among the first to examine tcVNS-induced autonomic changes over several hours rather than isolated time points, future research should assess how participants with highly variable responses contribute to overall trends in autonomic regulation. The relatively small sample size limits the statistical power of the study and may restrict the generalizability of the results to broader populations. This study did not stratify participants by sex, which may have influenced autonomic responses to tcVNS. Sex hormones, particularly estrogens and progesterone, regulate autonomic function via their effects on the HPA axis and vagal pathways (Dearing et al., 2022). Fluctuations in these hormones impact parasympathetic tone, stress reactivity, and baroreflex activity through receptors in key neuroendocrine and cardiovascular regions (Gurel et al., 2019). Future studies should assess sex-specific differences in tcVNS efficacy given that estrogens are cardioprotective and modulate autonomic homeostasis. Click or tap here to enter text.The expected attenuation of heart rate response in non-PTSD participants likely contributed to reduced statistical power for detecting significant effects of tcVNS in this group. Future studies should consider increasing the sample size of non-PTSD participants to better explore potential subtle effects in this population. Limited statistical power also restricted the complexity of the analyses performed (e.g., nesting of factors to test for PTSD vs. non-PTSD differences) and the interactions between factors that could be rigorously tested. This study also did not control for individual differences in sleep patterns or baseline anxiety levels, both of which could influence autonomic function and heart rate variability. Variations in sleep quality and pre-existing anxiety could have contributed to differences in physiological responses, potentially affecting the observed tcVNS effects. Future studies should consider assessing these factors to better isolate the specific impact of tcVNS on autonomic regulation. As an initial investigation into long-term cardiovascular effects, heart rate was the sole marker analyzed. Future work can expand on these efforts and explore other cardiovascular markers of interest to understand mechanisms and specific pathways of tcVNS’s effects better. This analysis is a secondary analysis; the initial goal of the clinical trial was not to investigate accrued effects of tcVNS. Hence, the findings of this study are limited to the setting of repeated stimulation over the course of 2–3 h. Future work can look to explore whether these effects accrue for longer and whether repeated stimulation is necessary for effects to accrue. While participants’ PTSD clinical histories were evaluated as detailed in Gurel et al. (2020a,b) to determine PTSD diagnosis and symptom severity, the results of these evaluations were not curated for subsequent reporting. The lack of specifics on each participant’s traumatic event is a limitation of this work, as the variability in traumatic events and length of time from the event may have influenced the responses observed. We also note that that while the effects of tcVNS on heart rate appear to build up over repeated stimulations, the precise mechanism behind this reduction remains unclear. It is also uncertain whether these effects represent a true accumulation over time or are due to a transient change after a certain number of stimulations. Further research is necessary to determine the nature of these effects and to establish their clinical relevance. In addition, it would be worthwhile to investigate whether sleep resets the physiological system or whether effects remain and linger onto the following day.

This study also kept participants supine throughout the entirety of the protocol, and participants were in a high-resolution positron emission tomography scanner throughout the protocol. These environmental effects may have moderated the differences we observed between active and sham heart rate following tcVNS. This remains to be investigated in studies incorporating multiple postures and/or environments. Future research is also necessary to elucidate the autonomic mechanisms underlying the accrued effects of tcVNS on heart rate.

The differences observed between PTSD and non-PTSD groups suggest that tcVNS may target specific pathological pathways unique to PTSD. These pathways might involve chronic dysregulation of the autonomic nervous system, heightened stress hormone levels, and altered neurocircuitry associated with fear and stress responses (Bottari et al., 2021; Liberati and Perrotta, 2024; Ogłodek, 2022; Pencea et al., 2020; Şahin Ekici et al., 2023; Timmer-Murillo et al., 2023). The selective efficacy of tcVNS in the PTSD group underscores the importance of understanding the distinct biological mechanisms at play in PTSD, which could lead to more tailored and effective interventions. Further research is warranted to elucidate these unique pathways and the precise mechanisms by which tcVNS exerts effects in PTSD patients that accrue over time.

## Conclusion

This study of traumatized individuals receiving active or sham tcVNS demonstrated that tcVNS reduces heart rate compared to sham stimulation, and that this reduction in heart rate significantly accrues over time for participants with PTSD. Notably, this accrued reduction in response to repeated tcVNS was observed over the course of a 2–3 h-long protocol. This indicates a temporary reduction of chronic cardiac arousal associated with PTSD in response to tcVNS. This reduction could be significant enough to provide protective effects against subsequent trauma recall, as was observed in this study where subsequent trauma recall increased heart rate for the active group back to its baseline level. This suggests that tcVNS may have autonomically protective effects for cardiac function in participants with PTSD in response to subsequent traumatic memories. This also seems to indicate that tcVNS could be a means to mitigate the chronic arousal and autonomic dysregulation associated with the disorder by potentially targeting pathways unique to those with the condition (Schneider and Schwerdtfeger, 2020). These speculations remain to be validated, but the findings encourage future work in the area to explore long-term autonomic effects using temporally precise analyses. Future studies should investigate whether repeated tcVNS use over days or weeks leads to sustained autonomic regulation and whether its effects extend beyond 24 h post-stimulation. Examining the feasibility of tcVNS as a long-term clinical therapy for PTSD would provide insight into its potential role in mitigating chronic autonomic dysregulation and associated cardiovascular risk factors. Notably, a strength of our work is the time series processing techniques used to observe heart rate dynamics at a second-by-second scale over an extended duration of 2–3 h. Analyzing the effects of tcVNS on heart rate in this way allowed us to have a more precise understanding of the time course of tcVNS effects on heart rate, which can be valuable in expanding our understanding of the underlying mechanisms of tcVNS’s effects on cardiovascular function.

## Data Availability

The data analyzed in this study is subject to the following licenses/restrictions: the participants of this study did not give written consent for their data to be shared publicly, so due to the sensitive nature of the research supporting data is not available. Requests to access these datasets should be directed to SS, ssundararaj06@gmail.com.
