# Supplementary figures and images for: Accrued reductions in heart rate following transcutaneous vagal nerve stimulation in adults with posttraumatic stress disorder
Source: Front Neurosci. 2025 Mar 28;19:1456662. doi: 10.3389/fnins.2025.1456662 (PMC11985822; doi:10.3389/fnins.2025.1456662)

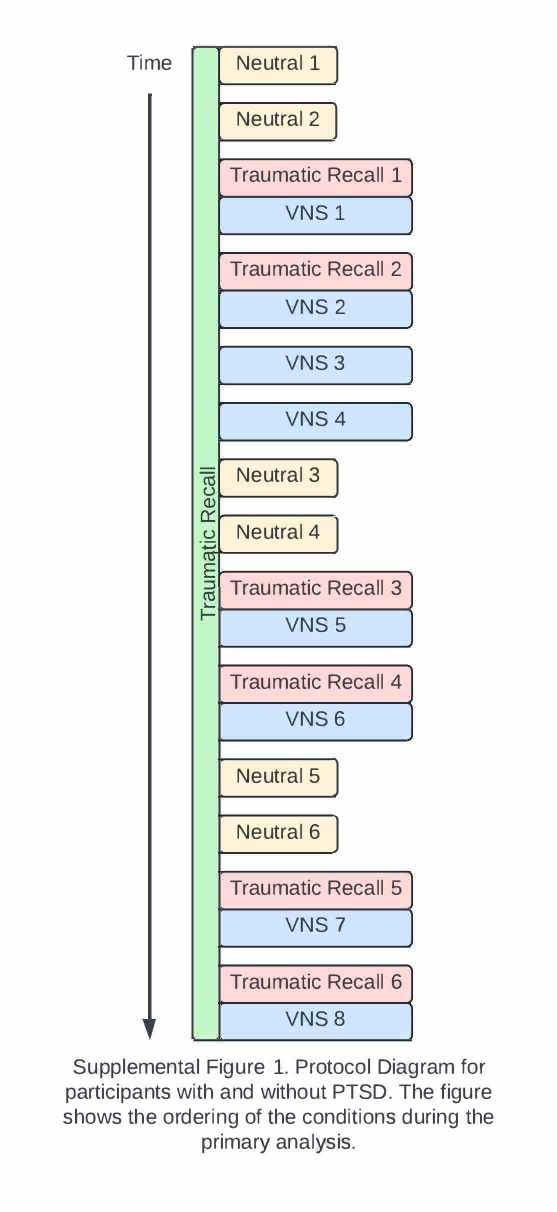

Supplement: Supplementary file 1 [file Image_1.png]
