# Supplementary material for: Accrued reductions in heart rate following transcutaneous vagal nerve stimulation in adults with posttraumatic stress disorder
Source: Front Neurosci. 2025 Mar 28;19:1456662. doi: 10.3389/fnins.2025.1456662 (PMC11985822; doi:10.3389/fnins.2025.1456662)
Supplement: Supplementary file 2 [file Table_1.DOCX]

| **Condition** | **Device Type** | **Mean Age (years)** | **Sex (M)** | **Sex (F)** | **Mean Height (cm)** | **Mean Weight (kg)** |
| --- | --- | --- | --- | --- | --- | --- |
| PTSD | Sham (11) | 42.54 ± 13.59 | 5 | 6 | 171.80 ± 13.59 | 85.5 ± 29.96 |
|  | Active (11) | 40.36 ± 12.64 | 1 | 10 | 168 ± 10.60 | 82.1 ± 27.51 |
| Non-PTSD | Sham (8) | 30.50 ± 3.51 | 3 | 5 | 170.80 ± 6.75 | 82.90 ± 14.81 |
|  | Active (11) | 34.45 ± 8.65 | 7 | 4 | 171.10 ± 14.87 | 76.10 ± 14.93 |

Supplementary Table 1. PTSD and Non-PTSD Participant Characteristics. Demographic characteristics of participants including the number of individuals of each sex, as well as the mean age, height, and weight for each condition (PTSD and non-PTSD) and device type (active tcVNS and sham) are reported.
