# Supplementary material for: Accrued reductions in heart rate following transcutaneous vagal nerve stimulation in adults with posttraumatic stress disorder
Source: Front Neurosci. 2025 Mar 28;19:1456662. doi: 10.3389/fnins.2025.1456662 (PMC11985822; doi:10.3389/fnins.2025.1456662)
Supplement: Supplementary file 3 [file Table_2.DOCX]

| Non-PTSD | | | | |
| --- | --- | --- | --- | --- |
| Condition | Sham Mean (SD) | Active Mean (SD) | Test Statistic | P-Value |
| Neutral 1 | 0.003 (0.015) | 0.013 (0.016) | t=-1.302 | 0.22 |
| Neutral 2 | -0.001 (0.024) | -0.003 (0.024) | t=0.157 | 0.877 |
| Trauma 1 | 0.025 (0.054) | 0.014 (0.073) | t=0.520 | 0.61 |
| Trauma 2 | -0.001 (0.074) | -0.004 (0.077) | t=0.122 | 0.905 |
| VNS 3 | -0.001 (0.044) | -0.020 (0.051) | t=0.707 | 0.496 |
| VNS 4 | -0.004 (0.045) | -0.021 (0.043) | t=0.705 | 0.496 |
| Neutral 3 | 0.018 (0.068) | 0.006 (0.095) | U=37 | 0.296_a_ |
| Neutral 4 | 0.007 (0.077) | 0.003 (0.075) | t=0.114 | 0.911 |
| Trauma 3 | 0.039 (0.072) | 0.027 (0.035) | t=0.316 | 0.756 |
| Trauma 4 | 0.020 (0.061) | 0.014 (0.036) | t=0.156 | 0.879 |

_a_ Mann Whitney U test was conducted instead of 2-sample t-test due to non-normal distribution

Supplemental Table 2. Statistical Analyses for Participants without PTSD. The mean active heart rate M (SD) and mean sham heart rate M (SD) for each condition are reported along with the corresponding p-values and test statistics.
